# The Role of Cysteine Cathepsins in Cancer Progression and Drug Resistance

**DOI:** 10.3390/ijms20143602

**Published:** 2019-07-23

**Authors:** Magdalena Rudzińska, Alessandro Parodi, Surinder M. Soond, Andrey Z. Vinarov, Dmitry O. Korolev, Andrey O. Morozov, Cenk Daglioglu, Yusuf Tutar, Andrey A. Zamyatnin

**Affiliations:** 1 Institute of Molecular Medicine, Sechenov First Moscow State Medical University, 119991 Moscow, Russia; 2 Institute for Urology and Reproductive Health, Sechenov University, 119992 Moscow, Russia; 3 Izmir Institute of Technology, Faculty of Science, Department of Molecular Biology and Genetics, 35430 Urla/Izmir, Turkey; 4 Faculty of Pharmacy, University of Health Sciences, 34668 Istanbul, Turkey; 5 Belozersky Institute of Physico-Chemical Biology, Lomonosov Moscow State University, 119991 Moscow, Russia

**Keywords:** cysteine cathepsins, cancer progression, drug resistance

## Abstract

Cysteine cathepsins are lysosomal enzymes belonging to the papain family. Their expression is misregulated in a wide variety of tumors, and ample data prove their involvement in cancer progression, angiogenesis, metastasis, and in the occurrence of drug resistance. However, while their overexpression is usually associated with highly aggressive tumor phenotypes, their mechanistic role in cancer progression is still to be determined to develop new therapeutic strategies. In this review, we highlight the literature related to the role of the cysteine cathepsins in cancer biology, with particular emphasis on their input into tumor biology.

## 1. Introduction

Cathepsins are lysosomal proteases and, according to their active site, they can be classified into cysteine, aspartate, and serine cathepsins [1]. Human cysteine cathepsins (Cts) are ubiquitously present in all organisms ranging from prokaryotes to mammals and have a highly conserved residue of cysteine in their active site. They are essential in the degradation of the proteins that are internalized in the lysosomes through endocytosis, phagocytosis, and autophagocytosis [2]. Furthermore, they are involved in the cellular protein digestion, zymogen activation, and extracellular matrix (ECM) remodeling [3,4]. Under physiological conditions, Cts are fundamental in maintaining tissue homeostasis, and they are involved in different processes such as immune response, apoptosis, development, and differentiation [5]. Alterations in Cts expression, localization, and activity have been associated with several pathological disorders, including cancer progression [6,7], and their ectopic expression is usually associated with poor prognosis [8]. 

Recent data have demonstrated that Cts localization is not limited to the endolysosomal compartment, but they were also found in the cell cytoplasm, nucleus, mitochondria, and extracellular space, indicating their broad biological activity [9,10,11]. In the context of cancerogenesis, secreted Cts contribute to the tumor ECM degradation and remodeling, while intracellular cathepsins are pivotal components of signaling pathways, which can enhance cancer cell growth and inflammation [12,13]. Also, Cts are engaged in response to anticancer therapy within the tumor microenvironment, and they can have crucial roles in the development of resistance phenomena to the therapeutics [14,15,16].

Here, we detail the relationship between human Cts and cancer, highlighting their involvement in tumor progression, infiltration, death, and their regulation in response to chemotherapeutics.

## 2. Cts Synthesis, Structure, and Localization

The family of Cts proteases includes the subtypes B, C, F, H, K, L, O, S, V, W, and X [17], which are synthesized as inactive zymogens and processed into their mature forms in the acidic environment of the lysosomes. In addition to the pH and the redox status of the surrounding microenvironment, their proteolytic activity is regulated by biological activators and inhibitors, such as cytokines, growth factors, collagen peptides, and endogenous inhibitors [18,19].

**Figure 1:**
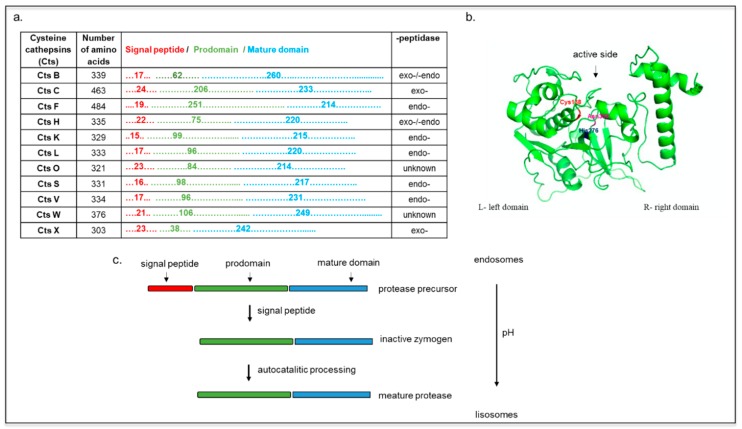
(**a**) Cysteine cathepsins (Cts) functional sequences. Representation of Cts according to their number of amino acids, length of domains (signal peptide, prodomain, and mature domain), and peptidase property. (**b**) Structure of cathepsin L. Cys138–His276–Asn300 triad at the active side is colored (model created with SWISS-MODEL and PyMOL). (**c**) Scheme Cts maturation as the function of endolysosomal pH. The mannose-6-phosphate pathway favors Cts delivery to endosomes, where they are eventually sorted into the lysosomes. At acidic pH, the pro-peptide is removed, and an active single-chain intermediate is generated. The removal of propeptide is mediated by other proteases (independent of autocatalytic activity). The single-chain molecule is further processed into mature two-chain form comprising an amino-terminal light chain and a carboxyl-terminal heavy chain.

According to their proteolytic activity, they can be further classified into exopeptidases (Cts C and X) or endopeptidases (F, O, S, K, V, L, and W), with Cts H and B possessing both endo- and exopeptidase activities (Figure 1a) [20]. 

Cts B, L, and H are expressed in most cell types, and they are involved in lysosomal nonspecific bulk protein degradation [21], while Cts S, K, V, F, C, and W show more tissue-specific expression and function [5,18,22]. All Cts are monomeric and single domain enzymes, and their structures are composed of two subdomains referred to as the L (left)- and the R (right)-domain, except for CtsC, which is present as a homotetramer (Figure 1b) [23,24]. In its native form, the Cts amino acid sequence includes a signal peptide, a propeptide, and a catalytic domain. The signal peptides can vary between 10 and 20 amino acids and mediate Cts translocation into the endoplasmic reticulum where, after they are cleaved, the inactive precursor is glycosylated [5]. These proteins are further transported to the Golgi apparatus, where the mannose oligosaccharides are phosphorylated, inducing their further translocation into the lysosomes via interaction with the mannose-6-phosphate receptor (M6PR) [25]. 

The propeptide sequences, which can range from 38 to 251 amino acids (Figure 1a) [21], have a pivotal role in regulating the folding of the catalytic domains, favoring Cts transport in the endo-lysosomal compartment and inhibiting the premature activation of the catalytic domain [26]. Exceptions to this process include CtsH, which is trafficked from the Golgi to the lysosome via the sortilin receptor [27]. The maturation of these zymogens occurs via autocatalytic cleavage at low pH for the endopeptidases (CtsH, L, S, K, V, F, B, and H), whereas the exopeptidases (CtsC and X) are processed by other endopeptidases, such as the CtsS and the CtsL (Figure 1c) [18,20,28]. The mature form of Cts contains a disulfide-linked heavy and light chain subunit, and it has an overall molecular weight that ranges from 20 to 35 kDa (except for CtsC that has a molecular weight of 50 kDa). Cts are usually localized in the acidic and reducing environment of the endo-lysosomal vesicles, but their expression and activity have also been detected in the cell nucleus, the plasma membrane, and cytoplasm. For example, CtsW is expressed in the endoplasmic reticulum of natural killer cells, and Cts K was found extracellularly and intracellularly in vesicles, granules, and vacuoles of osteoclasts and chondroclasts. In dendritic cells, Cts F is located in the Golgi apparatus of immature cells, while it is transferred to the endosomal/lysosomal vesicles in mature cells [29,30,31]. The nuclear localization of CtsL and localization of CtsB in plasma membrane correlated with metastatic tumors [32,33,34].

Also, most of them can be secreted into the extracellular compartment retaining their proteolytic activity (Table 1) [35,36].

### Inhibitors of Cysteine Cathepsins

Cts can be regulated by a variety of endogenous naturally occurring proteinaceous or small molecule inhibitors (i.e., aldehydes, ketones, nitriles, epoxysuccinyls [21]), which can interact with their active site reversibly or irreversibly [52]. Such a broadly distributed superfamily of protease inhibitors includes cystatins, thyropins, alpha2-macroglobulin, cytotoxic antigen 2β, and the members of the serpin family [17,53]. These endogenous inhibitors are classified into clans of stefins (family I), cystatins (family II), and kininogens (family III) structurally related to noninhibitory proteins, such as HRG (histidine-rich glycoprotein) and fetuins (family IV) [54]. The cystatins (100–120 amino acids) are mainly secreted from the cells, while the stefins (50–120 amino acids) and the kininogens (~120 amino acids) are the major cellular Cts inhibitors. Aberrant regulation of cystatin family members has been shown in several diseases, including cancer [55,56,57]. The misbalanced expression of Cts and their inhibitors can promote tumor growth, invasion, and metastasis. Stefins A and B can reversibly inhibit Cts B, H, S, L, and C while protecting the cell from the toxic effects of lysosomal enzymes leaked in the cell cytoplasm [58]. Stefin A and B were differentially demonstrated as suppressors and oncoproteins in many cases of human tumors (Table 2).

Interestingly, Stefin A lacks in a promoter and CpG islands (or any regions with high CpG density), suggesting that the *CSTA* gene may not be a target for DNA methylation, but the gene locus can experience loss of heterozygosity (LOH) for this Cts inhibitor [72,73]. Downregulation of Stefin B can be dependent on epigenetic factors, as demonstrated by northern blot, real-time PCR, and western blot analyses after treatment with 5-aza-2’-deoxycytidine [74].

The second class of cystatins includes cystatins C, E/M, F, D, S, SA, and SN with cystatin C and E/M representing the most expressed and investigated inhibitors. Cystatin C is expressed in a variety of human tissues (including kidney, liver, pancreas, intestine, stomach, antrum, lung, and placenta), and it can inhibit Cts B, L, S, H, and C activity [75,76]. Evaluation of cystatin C mRNA and protein expression showed no significant changes between human premalignant and malignant cells (brain, pituitary [77], renal carcinoma [78], breast [79], and colon cancers [80]), but a high level of cystatin C in the serum, the pleural effusions, and the ascites fluids collected from cancer patients was observed [81,82]. Decreased levels of cystatin C were detected in tissues of non-small lung cancer [83], squamous cell carcinoma of head and neck cancer [84], and glioblastoma [85]. On the other hand, the oncogenic effect of cystatin C was shown in null mice after injection with metastatic B16F10 melanoma cells and in an orthotopic model of breast cancer, where the depletion of cystatin C resulted in decreased growth of the tumor [86,87]. Its gene (*CST3)* contains a large CpG island (435bp) including 46 CpG dinucleotides that span the proximal promoter and exon 1, but no data reporting hypermethylation have emerged [74].

Cystatin E/M is usually expressed in a variety of human tissues and reversibly inhibits Cts B, V, and L. Low levels of this inhibitor were related to several kinds of cancers [55,74,88]. Its gene (*CST6)* is epigenetically silenced in cell lines derived from breast cancers (around 56% primary tumors and 85% of lymph node metastases) [89,90,91]. Here, significantly lower levels of cystatin E/M were observed when compared to healthy breast tissues. *CST6* promoter hypermethylation has been reported using bisulfite sequencing for 48% of neoplastic lesions analyzed [91]. In some cases, loss of cystatin E/M expression was not associated with *CST6* promoter hypermethylation, indicating that other potential mechanisms are at the base of the loss of Cystatin E/M expression. Alternatively, the beneficial effects of high expression of cystatin E/M were found in oropharyngeal squamous cell carcinomas [92].

Cystatin F is expressed in the cells of the immune system and synthesized as a dimer and activated by proteolytic cleavage. The monomeric form of cystatin F inhibits Cts F, K, L, V, S, and C [30,93]. Cystatin F was found in several human cancer cell lines, such as glioblastoma, colorectal carcinoma, and lung carcinoma cell lines, but also is overexpressed in tumor tissue of colorectal carcinoma compared to healthy tissues [94,95]. Although cystatin F expression has been mainly associated with antitumor immune responses, it was shown to favor metastatic spreading [94,96].

Cystatin D shows a restricted tissue distribution, being found in salivary submandibular and parotid glands, and it is an inhibitor of Cts H, L, and S [97,98]. Its suppressive effect in the tumor was shown in colorectal cancer cells, where low cystatin D expression correlated with epithelial-mesenchymal transition (EMT) and decreased expression of vitamin D receptor [99]. Interestingly, its expression increased after treatment of colon cancer cells with anti-tumorigenic vitamin D [97]. Cystatin D together with cystatins S, SA, and SN have a protective role in the host defense mechanisms against virus infection [100]. The upregulation of cystatin SN was demonstrated in several carcinomas, such as gastric [101], colorectal pancreatic [95], breast [102], and bone metastasis [103]. Increased cystatin SN expression in pancreatic carcinoma cells contributes to cell higher proliferation [102], and its knockdown consistently leads to in vitro and in vivo inhibition of tumor proliferation [104].

Taken together, current data indicate that the role of cystatins in cancer progression is complex, and they can potentially mitigate or enhance the cancer aggressiveness as a function of tumor phenotype and ancillary microenvironment conditions.

## 3. Cell Death

The endo/lysosomal compartment was shown to be involved in cell death, and many pieces of evidence support the hypothesis that some Cts could be involved in the regulation of apoptosis [105]. However, Cts positive or negative involvement in the cell death regulation depends on the cellular context, the Cts type [105,106,107,108], and, above all, on their release into the cell cytoplasm [109]. While apoptosis is frequently described as a result of caspase cascade activation, Cts can initiate, during the early phases of cell death, a possible caspase-independent process together with calpains and serine proteases [110,111]. CtsB, H, L, and S play an essential role in cleaving the classic caspase substrates, such as procaspase-1,-3,-7-8 [110,111], and in the release of pro-apoptotic factors such as cytochrome c from mitochondria [112,113], which leads to caspase activation and apoptosis. In this context, Cts were shown to target Bid (BH3 interacting domain death agonist) and/or degrade prosurvival Bcl-2 homologs, thereby triggering the pro-apoptotic activity of Bak (BCL2 antagonist/killer) and Bax (BCL2 associated X, apoptosis regulator) [114,115,116]. Moreover, XIAP (X-chromosome-linked inhibitor of apoptosis) was also found to be a target of Cts, suggesting that they can also mediate mitochondria caspase-dependent apoptosis [108]. 

The release of the Cts in the cell cytoplasm can occur through different mechanisms/factors including the activation of membrane receptors (such as tumor necrosis factor receptor 1 [113,117]) or the generation of reactive oxygen species (ROS) [118]. It was reported that the ligation of the tumor necrosis factor (TNF) receptor-1 by TNF-α in hepatocytes could cause the activation of caspase-8, which in turn labilizes the lysosomes favoring the release of CtsB. As a result of this phenomenon, mitochondrial release of cytochrome c (activating caspase-9 and eventually caspase-3) occurs, leading to apoptotic cell death [113]. The lysosome destabilization via ROS is regulated by an intraorganelle pool of redox-active iron, which sensitizes lysosomes to oxidative damage [119]. Furthermore, after UVA irradiation, the extracellular lysosomal contents are released and accompanied by translocation of lysosome-associated membrane protein-1 (LAMP-1) to the plasma membrane, which provides evidence for lysosomal exocytosis under stress conditions [120]. While new therapeutic interventions based on lysosomal permeabilization are under investigation to target apoptosis-resistant cancer cells [121], the release of the Cts into the cell cytoplasm can also be a result of chemotherapy [122]. In human hormone-refractory prostate cancer cells (HRPC), docetaxel treatment induced caspase-independent cell death through CtsB activity [123]. A similar mechanism was shown in non-small cell lung cancer (NSCLC) cells, where the microtubules stabilizing agent paclitaxel activates the release of CtsB via disruption of lysosomes in the cell cytoplasm, favoring cell death [124]. 

## 4. Autophagy

Autophagy is a lysosomal-dependent, intracellular self-digestion process in which damaged proteins and organelles are transported to the lysosome for degradation to maintain cellular homeostasis. Autophagy prevents cancer onset by removing damaged proteins and organelles, reducing ROS, and promoting the autophagic cell death. During apoptosis, Cts are released from the lysosomes into the cytoplasm, activating degradation cascades, whereas in autophagy, the lysosomes fuse with autophagosomes, forming the autophagolysosomes [125,126]. Alternatively, at very aggressive stages of tumor development, autophagy supports cancer development by protecting the malignant cells from cellular stress, supporting cancer progression [127]. Autophagy is initiated by the generation of a phagophore followed by autophagosomal formation and lysosomal fusion (autolysosome) [128]. Under stress conditions (e.g., hydrogen peroxide or starvation) the number of lysosomes decreases due to their continuous fusion with autophagosomes, which in turn increase [129]. This phenomenon is also responsible for clearing damaged lysosomes after permeabilization [130]. Recently it was shown that there is an active role of the Cts in the molecular switch between apoptosis and autophagy. In MCF-7-breast cancer cells, the treatment with the Cts inhibitor E64d increased autophagosome formation even when used in combination with the apoptosis inducer camptothecin [131]. The inhibition of CtsB and L was shown to favor autophagocytosis over apoptosis, where this process was accompanied by an accumulation of cellular stress and autophagic markers. The un-degraded proteins led to the induction of apoptosis, indicating that the balance between these two phenomena is controlled by many factors [132,133]. In neuroblastoma models, the inhibition of these two Cts resulted in significant accumulation in the cell cytoplasm of large cytoplasmic electron-dense vesicles and generation of multivesicular bodies, while in a double knockout model for *CTSB* and *L,* autophagic-like vacuoles accumulated over time [134]. Cts inhibition and consequent autophagocytosis were eventually linked to the dysregulation of the IGF-1 receptor/MAPK/Akt pathway, which is required for tumor cell growth and survival [135]. Interestingly, the inhibition of autophagy using Thymoquinone in glioblastoma cells induced CtsB leakage into the cytosol and caused activation of caspase-independent apoptosis [136], demonstrating that these phenomena are strictly linked to each other. 

We can summarize that Cts may be key regulators of pro-survival autophagy in cancer cells, and Cts inhibition could increase the efficacy of cancer treatment focused at inhibiting autophagy. On the other hand, lysosomal destabilization could inhibit the metabolization of substrates generated through autophagy, prevent clearance of ROS, and increase Cts release in the cell cytoplasm where their activity can amplify the apoptotic signaling.

## 5. Tumor Matrix Cellular Degradation

The EMT is considered an essential process at the base of tumor cell motility and invasiveness. EMT is triggered by multiple transcriptional and biochemical cascades, and it results in the acquisition of migratory and invasive properties typical of mesenchymal cells [137]. 

In this context, the CtsL is involved in EMT, and its overexpression in lung cancer cells leads—via nuclear factor kappa B (NF-κB) signaling—to the upregulation of nuclear factors belonging to the snail family transcriptional repressors Snail and Slug, and the zinc finger e-box binding homeobox proteins ZEB1 and ZEB2 [138]. Also, Snail can promote the nuclear translocation of CtsL where it degrades its substrate homeobox protein Cux-1, leading to the inhibition of E-cadherin expression and the induction of Snail transcription that promotes EMT [139]. Similar observations were described in cell lines derived from lung and breast cancer (A549 and MCF-7, respectively), where EMT induced by TGF-β activation was associated with the increased protein expression of CtsL and Snail via phosphatidylinositol 3-kinase (PI3K)-AKT and Wnt signaling pathways. Additionally, *CTSL* knock-down in A549 cells favored mesenchymal to epithelial transition (MET) in vivo, inhibiting xenograft tumor growth [138,140]. EMT regulation by CtsB is connected via the E-box element regulation, which controls EMT activator factors [141]. Besides, it was shown that the Cts B expression knock-down was followed by an increase in Cts X expression compensating the absence of this protease [142,143]. Cts X overexpression was also associated with EMT of hepatocellular cancer and with the upregulation of matrix metalloproteinases (MMP2, MMP3, and MMP9) [144].

In this scenario, Cts cover a central role in tumor progression when they are released in the ECM. They can be secreted or associated with the plasma membrane and caveolae [145]. When secreted, Cts can favor cancer cell spreading by modifying and degrading the ECM and/or activating the matrix metalloproteinases (MMPs) and urokinase plasminogen activator [146,147]. Also, the acidic extracellular environment of the tumor is ideal for favoring the proteolytic activation of the Cts when they are secreted [146,148]. It was demonstrated that ECM degradation by the active form of Cts B and X increased the release of growth factors, including TGFβ-1, an EMT activator [144,149], while regulating cell adhesion. 

In the context of the tumor neo-angiogenesis and cell–cell communication, cancer cells can stimulate endothelial cells through two main ways: (1) in a direct manner via secretion of soluble factors and expression of adhesion receptors, gap junctions/tunneling nanotubes, and microvesicles (MVs) [150,151,152]; and (2) in an indirect manner by secretion of proteases into the extracellular space or by changing the pH [148,153,154,155]. One of the causes of resistance to anti-angiogenic therapies has been attributed to the ability of cancer cells to communicate with endothelial cells [154]. Lysosomal exocytosis of Cts such as B, H, and L can regulate this phenomenon in various tissues [7,156,157,158]. For example, the increased secretion of CtsL via lysosome exocytosis was correlated with enhanced tumor cell migration and invasion [7]. HT 1080 cells were shown to secrete CtsL directly into the medium through a lysosome exocytosis independent pathway, and the secreted protein was approximately 10-fold more active (32 kDa) than that detected in the cells [159]. 

Inhibition of the gene expression of Cts B in glioblastoma cells attenuated their migration and invasion while reducing cell–cell interaction in human microvascular endothelial cells in both in vitro and in vivo models [160]. 

The acidification of the tumor microenvironment has been shown to increase invasiveness and metastasis, suggesting that it offers a favorable advantage for cancer development, while it is toxic for healthy stromal cells [161]. Also, tumor acidic cell environment increases the bioavailability of many growth factors (e.g., VEGF) by inducing MVs rupture in the microenvironment [162], also favoring the release of CtsB [162], as shown in ovarian, breast, and colon carcinoma three-dimensional (3D) cultures [148,163,164]. The remodeling of the ECM is critical for the tubulogenesis of endothelial cells during cancer vascularization. CtsB was found in human umbilical vein endothelial cells to co-localize within intracellular vesicles and caveolae and participate in cell polarization and migration via secretion of MVs [163]. It was reported that active CtsS controls the production of matrix-derived angiogenic factors such as type IV collagen, canstatin, arresten, and tumstatin [165]. The anti-Cts S antibody Fsn0503 also potentiated the antiangiogenic effects of anti-VEGF therapies [166]. 

CtsL plays a crucial role in the invasive and the functional capacities of endothelial progenitor cells (EPC) in the EPC-mediated neovascularization process [167]. Gene expression analysis on breast tissues revealed significant upregulation of *CTSL* and strong correlation with increased relapse and metastatic incidence. Silencing of *CTSL* using shRNA or KGP94 (a small inhibitor of CtsL) treatment led to a significant reduction in MDA-MB-231 cells to induce angiogenesis in vivo. Moreover, the analyses with KGP94 in vitro demonstrated a considerable decrease in angiogenic properties such as cell sprouting, migration, invasion, tube formation, and proliferation [7]. CtsK and Cts B are essential for extracellular components, such as type I and IV collagens or elastin [168,169], and many studies demonstrated the critical role of CtsK and Cts B in neovascularization [170,171]. The increase of CtsX expression in tumor cells and tumor-associated immune cells is associated with progression and metastasis of gastric cancer, prostate cancer, hepatocellular carcinoma, and malignant melanoma [144,172,173,174]. CtsX modulates cell adhesion and migration by modulating the interaction with integrin receptors, thus supporting invasion through the ECM [175,176]. 

## 6. Crosstalk between Cell Death, Autophagy, and Tumor Matrix Degradation

**Figure 2:**
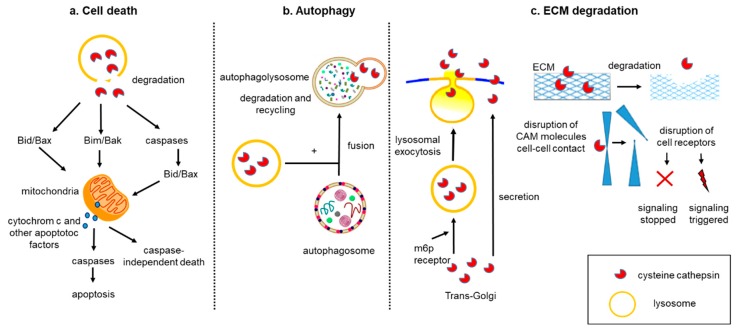
Role of Cts in cell death, autophagy, and ECM degradation. (**a**) Cts are released into the cytosol and are involved in apoptotic pathways. The first pathway includes a direct cleavage of Bid and/or Bak/Bim, translocation of these pro-apoptotic proteins to the mitochondrial outer membrane, which induces the release of apoptogenic factors such as cytochrome c and subsequent activation of downstream caspases and apoptosis. The second pathway involves a direct cleavage of caspases followed by cleavage of Bid and/or Bax, translocation of these proteins to mitochondria, and similar downstream events leading to apoptosis. The third option is independent-caspase apoptosis. (**b**) Upon induction of autophagy, cytoplasmic materials are sequestered by a double-membraned structure, autophagosome, which fuses with lysosomes to become autolysosomes. In autolysosome, the sequestered cargos are degraded and recycled. (**c**) Within the Golgi, Cts are transported into lysosomes via the mannose-6-phosphate (m6p receptor) pathway, but a minor population of Cts (~5%) is not converted to the m6p form and as a result is shunted into the exocytosis pathway [5]. Additionally, this direct secretion pathway of CtsL was detected in human fibrosarcoma cells (HT 1080) [159]. In the extracellular space, Cts cleave different targets. e.g., cell adhesion molecules (CAM), cell–cell contacts, and proteins of ECM, which affect cell adhesion and migration. Proteolytic products of these cleavages can act as signaling molecules and influence growth and invasion of cancer. Next, the cleave of receptors by Cts can result in either constantly triggered or inhibited signalings.

The aforementioned biological processes (cell death, autophagy, and tumor matrix degradation) may synergistically contribute to a cancer drug resistance. While chemotherapeutics were designed to induce apoptosis, their prolonged administration can increase the chance to develop drug resistance phenomena. In this scenario, the induction of autophagic death can be a promising approach to overcome cancer drug resistance. [177]. Autophagy and apoptosis are catabolic pathways essential for homeostasis, and usually they are both considered tumor suppressors. Whereas autophagy is a self-degradative process removing misfolded/aggregated proteins and degraded organelles, apoptosis is the canonical programmed cell death. Several molecular pathways interconnect autophagy and apoptosis, thus any misregulation of these processes can favor cancer cell proliferation. However, autophagy plays a dual role in cancer, as it has recently been shown that this process can also facilitate the survival of tumor cells in stress conditions such as hypoxia or low-nutrition environments [178]. Cts are engaged in all of these biochemical pathways, and they can alternatively favor or inhibit tumor growth. The material destined for degradation enters the lysosomes primarily via endocytosis, autophagy, and phagocytosis and is degraded through the action of hydrolases, including Cts [13]. In autophagy, the functional integrity of the lysosomal compartment provides active degradation, which can counteract apoptosis. In situations when Cts are released into the cytosol upon lysosomal membrane permeabilization, they can amplify the apoptotic signaling or initiate the lysosomal pathway of apoptosis via Bid and/or Bak/Bim cleavage. [179] (Figure 2a,b).

The malignant phenotype of cancer cells is accompanied by ECM degradation and morphological changes, which are typical events characterizing EMT. As a result of this transformation, cells modulate the profile of adhesion proteins, cell receptors, cytoskeleton polarization, and secretion of molecules, such as cytokines, growth factors, and proteases (Figure 2c) [180]. All these events can upregulate cancer motility and invasion properties of tumor cells. Overexpression of primary factors such as Twist, Zeb, Slug, and the activation of signaling pathways such as Hedgehog and TGFβ/Smad4 is induced during EMT and correlates with the onset of drug resistance phenomena [181]. Furthermore, the metastatic spreading relies on newly formed vessels, which are accompanied by proteolytic degradation of the endothelial basement and ECM [182].

## 7. Drug Resistance

Cancer drug resistance can have many different origins, such as drug inactivation, increased or decreased expression of efflux and influx pumps, respectively, modulation of specific mechanisms of cell death, autophagy, the occurrence of EMT, aberrant cell–cell communication, and epigenetic alterations, amongst others. These phenomena can occur individually or concurrently while affecting the efficacy of a wide range of cancer therapeutics [177]. One of these phenomena is strongly connected to the biology of the lysosomes, since many of the classical chemotherapeutics are sequestered in the acidic pH lumen of these organelles [183], reducing their ability to interact with their targets. It was shown that the amount of drug accumulating in the lysosomes is directly proportional to the cellular tolerance against the cytotoxic effects of the therapeutic [184,185]. Weakly basic, hydrophobic anticancer drugs after internalization may undergo rapid incorporation into lysosomes. Upon entrapment in lysosomes, lysosomotropic compounds appear to inflict various deleterious effects on the membrane of the lysosomes, provoking lysosomal recycling, biogenesis, and exocytosis. As a result of this phenomenon, the lysosomal sequestration of chemotherapeutic drugs in lysosomes prevents them from reaching their intracellular target sites [183]. This was observed for several classical chemotherapeutics, including Doxorubicin [186], Mitoxantrone [187], Sunitinib [188] and Daunorubicin [189], where their internalization was followed by their accumulation in the lysosomes [190]. 

Another mechanism of drug resistance is related to lysosomal exocytosis, a process in which lysosomes are recruited to the cell surface and fuse with the plasma membrane via a Ca^2+^-dependent process releasing their cargo (and the entrapped drug) into the extracellular milieu [191]. This phenomenon occurs as an adaptation of cells to the hypoxic and the acidic environment of the tumor. Hypoxia leads to the acidification of the tumor microenvironment, which can trigger proteases secretion, including Cts, from cancer cells to the extracellular space, enhancing the tumor invasiveness [192]. Interestingly, it was shown that oral administration of buffer, which neutralized intratumoral pH, reduced metastasis of cancer cells that was accompanied by lower CtsB activity [193]. 

The specific contribution of Cts in the occurrence of chemoresistance phenomena is currently under debate. The overexpression of CtsB and L was associated with increased drug inactivation and, above all, with increased lysosome trafficking to the plasma membrane and secretion of lysosomal cargo [194]. As a result of this phenomenon, *CTSL* knock-down in ovarian cancer cells SCOV3 resulted in increased apoptosis induced by paclitaxel (the most common drug in the management of ovarian cancer) [16]. Also, Cts L played a role in inhibiting the cell senescence process in different tumor cell lines, providing an additional mechanism of protease-mediated drug target elimination (trapping a drug in lysosomes) and drug resistance [15,138,195]. Treatment of drug-resistant neuroblastoma cells with doxorubicin and pan-caspase inhibitor (Q-VD-OPH) significantly decreased cell viability and senescence phenotype, as evidenced by an increased p21/WAF1 expression, senescence-associated β-galactosidase activity, and cell growth arrest. Experiments have established that this phenomenon was the result of CtsL inhibition [15]. 

In this context, Cts L inhibition increased Doxorubicin cell accumulation and a more favorable nuclear distribution of the drug in the cells despite P-gp expression [195]. The CstL inhibition stabilized and enhanced the availability of cytoplasmic and nuclear drug targets such as estrogen receptor-α, Bcr-Abl, topoisomerase-IIα, histone deacetylase 1, and the androgen receptor I, increasing the cellular response for different therapeutics drugs (doxorubicin, tamoxifen, imatinib, trichostatin A, and flutamide) [195].

These findings provide evidence for the potential role of Cts as targets, and their inhibitors can represent a tool to suppress cancer resistance to chemotherapy and increase their sensitivity to the drugs.

## 8. Perspectives

The role of Cts in tumorigenesis was demonstrated around 40 years ago [196]; recently, they were detected in the cytoplasm, the nucleus, the mitochondria, and the ECM of different tumor cells, highlighting their importance in cancer progression. 

Currently, inhibitory antibodies against CtsS tested in preclinical investigations show a significant reduction of tumor growth and improved chemotherapeutic efficiency [197,198]. Moreover, Cts could represent optimal diagnostic [199] and prognostic [200] markers, and they can be exploited for designing enhanced drug delivery approaches [201]. Cts can be used to favor a payload release conjugated to nanotherapeutics via a peptide link sensitive to their action or to activate prodrugs [202,203]. Cts B and L were proposed as biomarkers for cancer detection, and they were described as the valuable markers for lung [204], colon [205], and ovarian cancers [206], where their expression is usually inversely correlated with patient outcomes. Several approaches have been developed to block Cts activity, including small-molecule inhibitors and antibodies. The downregulation of CtsB using synthetic inhibitors such as CA-074 inhibited the neovascularization and the formation of bone metastases in breast cancer [207].

However, medical applications are more focused on targeting extracellular Cts, while intracellular Cts are considered as key players in complex signaling pathways such as autophagy and cell death. Cts in the extracellular space are released by both tumor cells and stromal cells, including tumor-associated macrophages, myoepithelial cells, and fibroblasts. In this scenario, Cts released by the macrophages can stimulate the release of interleukins and cytokines, which favor the inflammation. Interestingly, CtsB, H, and S secreted by macrophages in pancreatic neuroendocrine cancer reduced progression by increased apoptosis and reduced angiogenesis [14]. On the other hand, CtsL secreted by ovarian cancer cells mediated the tumor progression [208]. In this context, the beneficial and the harmful effect of cell-specific, extracellular Cts still need to be investigated. 

## 9. Conclusions

Many conflicting pieces of evidence demonstrate that changes in Cts expression and in the expression of their natural inhibitors could, in specific contexts, both favor and inhibit cancer growth and spreading. According to these observations, the mechanisms regulated by different types of Cts and their isoforms should be strictly defined for cancer type and tumor stages, while their potential utility as pharmacological targets needs more investigation. Finally, despite the deep investigation performed on Cts B, S, K, and L, the potential involvement of other Cts in cancer disease still needs to be unveiled, hopefully through the development of new molecular specific inhibitors and knock-down in vitro and in vivo models, which are currently lacking in the field.

## Figures and Tables

**Table 1:** Extracellular Cts and their extracellular matrix (ECM) substrates.

Cysteine Aathepsins (Cts)	Extracellular Localization	ECM Proteins Degraded by Cts	References
**CtsB**	+	aggrecan, proteoglycan, collagen I, II, IV, IV, IX, X, XI, laminin fibronectin, osteocalcin, osteonectin	[37,38,39,40,41,42,43]
**CtsC**	N/A	N/A	-
**CtsF**	+	proteoglycan	[44]
**CtsH**	+	osteocalcin	[42]
**CtsK**	+	aggrecan, elastin, osteonectin, collagen I, II	[45,46,47]
**CtsL**	+	proteoglycan, aggrecan, collagen I, II, IX, XI, fibronectin, laminin, osteocalcin	[38,39,42,45,48,49]
**CtsO**	N/A	N/A	-
**CtsS**	+	aggrecan, proteoglycan, collagen, elastin, fibronectin, osteocalcin	[3,38,45]
**CtsV**	+	elastin	[50]
**CtsW**	N/A	N/A	-
**CtsX**	+	N/A	[51]

**Table 2:** Role of stefins in human cancers.

Stefin	Cancer Type	Function	References
**Stefin A**	Breast	The low expression level is associated with cancer development and aggressiveness	[59,60]
	Brain		[61]
	Esophageal squamous		[62,63]
	Lung		[64]
	Prostate		[65]
**Stefin A**	Breast	The low expression correlates with a better outcome of patients	[66]
	Liver		[67]
	Brain		[68]
**Sfefin B**	Colorectal	The low expression level is associated with cancer development and aggressiveness	[36]
	Breast		[69]
	Head and neck		[63]
**Stefin B**	Liver	The low expression correlates with a better outcome of patients	[67]
	Ovarian		[70]
	Brain		[68]
	Bladder cancer		[71]

## Abbreviations

CTS	Cysteine Cathepsins
LOH	Loss of Heterozygosity
BID	BH3 Interacting Domain Death Agonist
BAK	BCL2 Antagonist/Killer
BAX	BCL2 Associated X, Apoptosis Regulator
XIAP	X-Chromosome-Linked Inhibitor of Apoptosis
ROS	Reactive Oxygen Species
TNF	Tumor Necrosis Factor
LAMP-1	Lysosome-Associated Membrane Protein-1
HRPC	Hormone-Refractory Prostate Cancer
NSCLC	Non-Small Cell Lung Cancer
E-64-d	Protease Inhibitor
EMT	Epithelial–Mesenchymal Transition
MET	Mesenchymal-Epithelial Transition
SNAIL1	Snail Family Transcriptional Repressor 1
SLUG	Snail Family Transcriptional Repressor 2
ZEB	Zinc Finger E-Box Binding Homeobox
NF-κB	Nuclear Factor Kappa B
PI3K	Phosphatidylinositol 3-Kinase
MMP	Matrix Metalloproteinase
ECM	Extracellular Matrix
MV	Microvesicle
KGP94	Cathpesin L Inhibitor
